# The Relationship between Tooth-Ache and Neuralgia

**Published:** 1912-07-15

**Authors:** E. Tromner

**Affiliations:** Neurologist, Hamburg


### THE RELATIONSHIP BETWEEN TOOTH-ACHE AND NEURALGIA.

BY DR. E. TROMNER, NEUROLOGIST, HAMBURG.

In addressing the Hamburg Dental Association on this subject, Dr. Tromner urged the necessity of a closer inter-relationship between dental and general medical treatment.

From being a step-mother medicine has become the foster mother of dentistry. But the exchange hitherto has not been fairly balanced. Dentistry has profited enormously by the study of general medicine, but medicine is still hampered by an inadequate recognition of the bearings of dentistry on medical treatment. The relationship between dentistry and neurology is especially significant. The subject of the neurology of the teeth is generally exhausted when one has considered caries, non-eruption, periostitis, the formation of secondary dentine as causes of trigeminal neuralgia, anomalies of position as signs of degeneration and atrophy of the aveolar process as part of the development of Tabes dorsalis. When diseases of the nerves are considered as a whole, all disturbances of the peripheral and central nervous system must be included, nor must mental diseases be left out —and in this branch of medicine, in neurology, although the *role* of dental troubles is a limited one, yet in cases of chronic disorder, either of development or of the nerves directly affecting the teeth, they must be taken into consideration— and in the matter of diagnosis it does not seem impossible, when one considers the work of Schafer, that there shou'd come a day when many diseases that affect the body can be ascertained by an examination of the mouth.

In any case the anomalies of the teeth have a three-fold significance for the nervous system. They may be (1) symptoms or indications of deep-seated neurotic or neuropathic conditions, (2) the direct cause of nervous disorders, or (3) the results of such disorders.

It is true that there are men of genius with poorly developed teeth and dunces with perfect ones, but faulty dentition generally points to a whole series of defects. Epignathism and the presence of adenoids generally means limited mental development.

But with regard to caries we cannot pronounce with any certainty. It is not established that neurotic subjects have on the whole worse teeth than others, and a seriously carious mouth cannot be counted as a sign of degeneration.

Of much greater importance is the *role* of the teeth in causing nervous trouble; difficult eruption may induce a whole series of disturbances of the nervous system.

In treating trigeminal neuralgia doctors are apt to overlook the importance of a thorough examination of the condition of the mouth —on the other hand, it must be recognized that there is a great deal of facial neuralgia that is quite unrelated to teeth, and a fuller recognition of this fact on the part of the dental profession would save many teeth which are sacrificed to no purpose.

It is not an uncommon experience with the writer to discover pyorrhea alveolaris, a chronic catarrh of the sockets of the teeth, developing as a result of a generally depressed condition of the organism. There can be no doubt that a careful research into these matters, carried on reciprocally between dentists and medical practitioners, would bring to light and to solution very many slumbering problems.—Abstract from the *Deutsche Monatsschrift fur Zahnheilkunde.*

